# Case Report: SMARCA4-deficient NSCLC with brain metastasis harboring co-mutations in chromatin remodeling and DNA damage repair pathways

**DOI:** 10.3389/fonc.2025.1683301

**Published:** 2025-11-27

**Authors:** Jiaqin Song, Shikun Yang, Lei Xia

**Affiliations:** 1 Department of Cancer Center, The Second Affiliated Hospital of Chongqing Medical University, Chongqing Key Laboratory of Immunotherapy, Chongqing, China; 2 Department of Pathology, The Second Affiliated Hospital of Chongqing Medical University, Chongqing, China

**Keywords:** SMARCA4, chromatin remodeling genes, DNA damage repair (DDR) genes, genomic profiling, non-small cell lung cancer, precision therapy

## Abstract

SMARCA4 (SWI/SNF-related matrix-associated actin-dependent regulator of chromatin subfamily A member 4) is the core ATPase subunit of SWI/SNF chromatin remodeling complexes. Its deficiency constitutes a rare and aggressive subtype of non-small cell lung cancer (SMARCA4-DNSCLC) characterized by rapid progression, propensity for early metastatic dissemination, and dismal prognosis (median overall survival: ~6 months). Notably, SMARCA4 mutations demonstrate significant co-occurrence with DNA damage repair (DDR) pathway dysregulation, though the clinical implications and molecular interplay of these co-mutations remain poorly understood. We present a treatment-naïve SMARCA4-DNSCLC case with synchronous brain metastasis harboring a unique genomic profile: concurrent mutations in chromatin remodeling genes (SMARCA4, CHD8, NSD1) and DDR pathway genes (ATR, BARD1, TP53), accompanied by elevated tumor mutational burden (TMB-H). This molecular signature implies potential synergistic effects between chromatin instability, compromised DNA damage repair mechanisms, and augmented immunogenicity. Through comprehensive genomic analysis, we elucidate the biological significance of this mutational landscape and discuss its therapeutic implications, aiming to advance precision diagnosis and guide innovative treatment strategies for SMARCA4-DNSCLC.

## 1 Introduction

Based on relevant research, lung cancer continues to pose a significant global health burden, with its incidence remaining high. Non-small cell lung cancer (NSCLC) is the most predominant pathological type, accounting for approximately 85% of all lung cancer cases (1). Within NSCLC, adenocarcinoma and squamous cell carcinoma are the most common subtypes. In recent years, the treatment landscape for NSCLC has witnessed remarkable progress, particularly with breakthroughs in targeted therapy and immunotherapy, which have profoundly transformed the therapeutic paradigm (2). Targeted agents against driver genes such as EGFR, ALK, ROS1, and BRAF have significantly improved outcomes for specific patient subgroups, while immune checkpoint inhibitors (e.g., anti-PD-1/PD-L1 antibodies) have greatly expanded treatment options for advanced-stage patients and provided long-term survival benefits for some. Furthermore, effective inhibitors targeting emerging targets like the KRAS G12C mutation have been successfully applied in clinical practice (3). Notably, a distinct molecular subtype defined by SMARCA4 DNSCLC - has been identified within NSCLC. These tumors are typically highly aggressive, respond poorly to conventional chemotherapy, and are associated with a poor prognosis. They often co-occur with other genetic alterations (e.g., KEAP1, STK11), presenting unique challenges and opportunities for current clinical management and novel drug development (4).

SMARCA4 is a tumor suppressor gene located at 19p13.2 and encoding the BRG1 protein, which is one of the important subunits of the SWI/SNF chromatin remodeling complex and is considered to be a powerful regulator of transcription and DNA repair, thus playing an important role in cell cycle control and cell differentiation (4). As a tumor suppressor aberrantly expressed in approximately 10% of NSCLC, SMARCA4-DNSCLC exhibits a unique combination of histomorphological, immunophenotypic, and molecular genetic attributes with weak response to conventional chemotherapy and poor prognosis (5). In lung cancer patients, SMARCA4 gene mutations do not always occur alone and are often combined with gene mutations such as TP53, KRAS, KEAP1, and STK11 (6). Studies have found that SMARCA4 combined with TP53 mutation may indicate a specific tumor subtype or a more aggressive tumor phenotype (7), which is helpful to classify and diagnose tumors more accurately and provide a basis for subsequent precise treatment. However, at present, the clinical and pathological characteristics of such combined mutations are not completely clear.

In this paper, we report a case of simultaneous mutations in multiple chromatin remodeling factors (SMARCA4, CHD8, NSD1) and DDR genes (ATR, BARD1, TP53), as well as TMB-H. This typical case has significant implications for both pathologists and clinicians. This case report further highlights the complex scenario of lung cancer subtypes and highlights the importance of a nuanced understanding of their molecular basis to guide accurate diagnosis and tailored therapeutic approaches.

## 2 Case presentation

**Figure 1:**
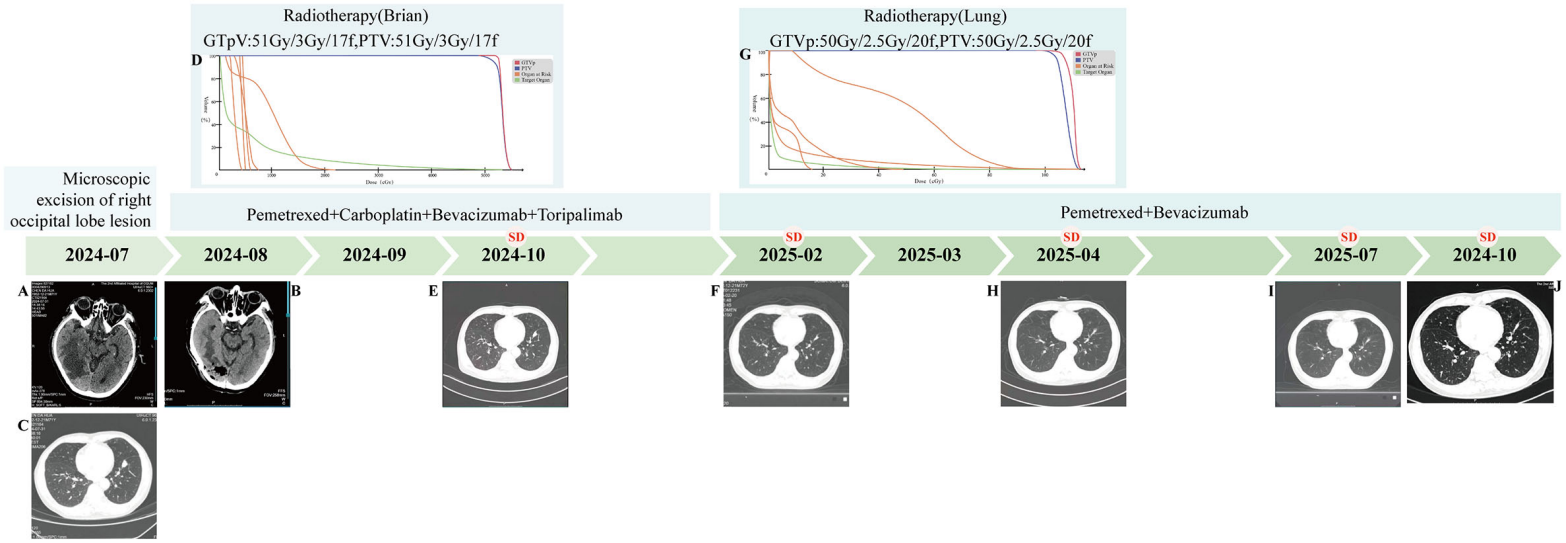
Patient Treatment Flowchart. **(A)** (2024-07) Mass shadow seen under the right occipital inner table with clear boundary and larger cross section of about 35*30mm. **(B)** (2025-08) Right occipital inner table with adjacent tissue edema and postoperative manifestations. **(C)** (2024-07) Solid nodule in the anterior-inferior basal segment of the left lower lobe of the lung (size about 22.7mm*14.7mm), with burr and lobulation visible at the margin. **(D)** (2024-09) Radiotherapy (Brain) DVH map. **(E)** (2024-10) Solid nodule in the anterior medial basal segment of the left lower lobe of the lung (size about 19.1mm*11.1mm). **(F)** (2025-02) Solid nodule in the anterior medial basal segment of the left lower lobe of the lung (size about 18.6mm*5.1mm). **(G)** (2025-03) Radiotherapy (Lung) DVH map. **(H)** (2025-04) Solid nodule in the anterior medial basal segment of the left lower lobe of the lung (size about 16mm*7mm). **(I)** (2025-07) Solid nodule in the anterior medial basal segment of the left lower lobe of the lung (size about 17.8mm*7.9mm). **(J)** (2025-10) Solid nodule in the anterior medial basal segment of the left lower lobe of the lung (size about 18.4mm*7.3mm).

**Figure 2:**
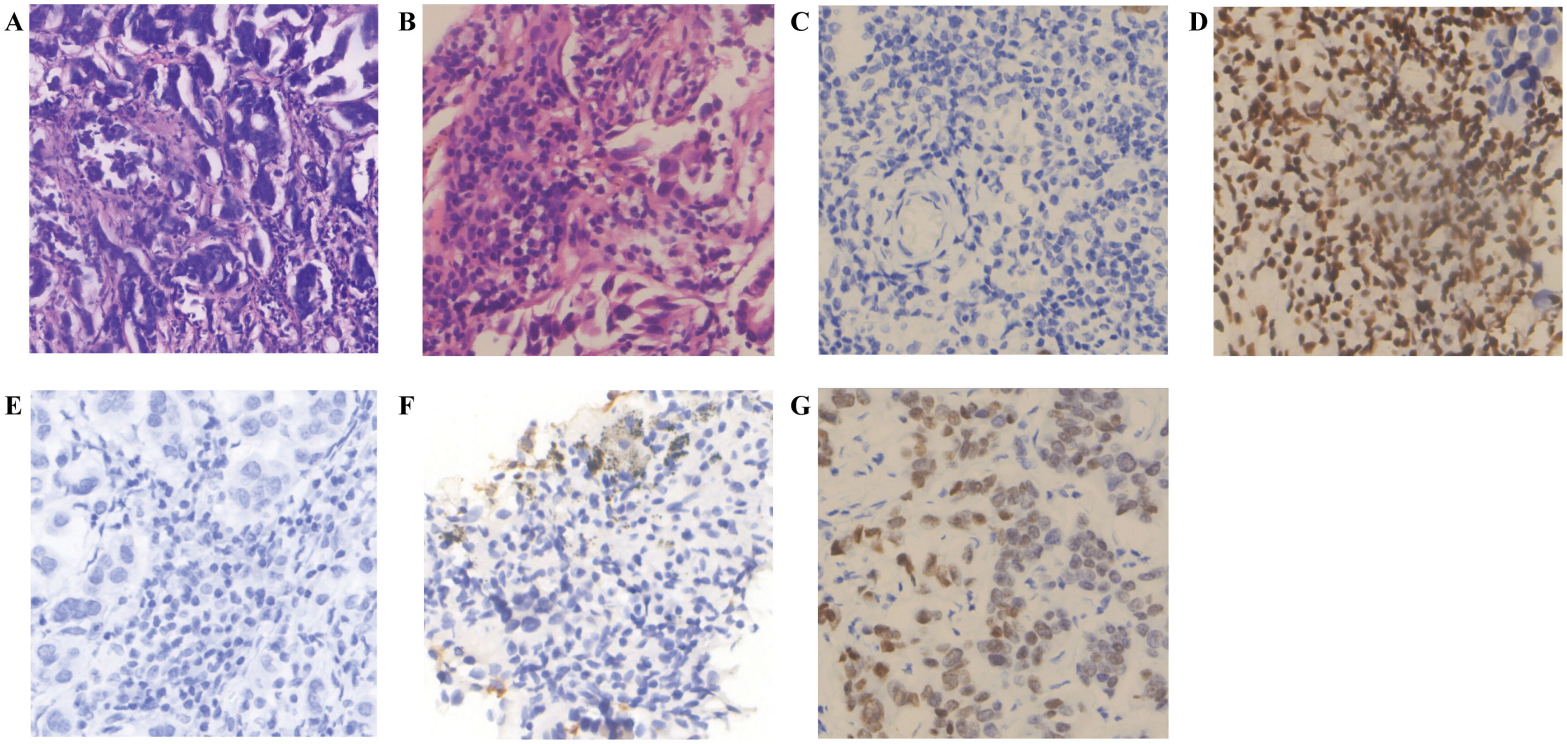
Immunohistochemistry of brain and lung tumor samples. **(A)** H&E staining (Brain tumor sample) (Magnification, ×20; Scale bar, 100μm). **(B)** H&E staining (Lung tumor sample) (Magnification, ×40; Scale bar, 50μm). **(C)** BRG-1 (-) (Brain tumor sample) (Magnification, ×40; Scale bar, 50μm). **(D)** BRG-1 (-) (Lung tumor sample) (Magnification, ×40; Scale bar, 50μm). **(E)** NapsinA (-) (Brain tumor sample) (Magnification, ×40; Scale bar, 50μm). **(F)** NapsinA (+) (Lung tumor sample) (Magnification, ×40; Scale bar, 50μm). **(G)** TTF-1 (+) (Brain tumor sample) (Magnification, ×40; Scale bar, 50μm).

Patient, male, 71 years old, with a history of smoking for 50 years at an average of 10 cigarettes per day, was admitted in July 2024 due to “vision decline for 4 months.” Head computed tomography (CT) revealed a mass under the inner plate of the right occipital lobe, with clear boundaries and a maximum diameter of approximately 35 × 30 mm (**Figure 1A**). Nuclear medicine tests (lung cancer markers: specific serum tumor markers) showed elevated levels: carbohydrate antigen-724 > 300.00 U/ml, carbohydrate antigen-125 at 245.33 U/ml, and carcinoembryonic antigen at 688.03 ng/ml. After completing preoperative examinations, the patient underwent “microscopic resection of the right occipital lobe lesion” in August 2024. Postoperative immunohistochemistry indicated: Ki67 (+, 30%), CK7 (+), Vimentin (-), CK5/6 (-), P40 (-), CK (Pan) (+), BRG-1 (-) (**Figure 2C**), NapsinA (-) (**Figure 2E**), TTF-1 (+) (**Figure 2G**), CK20 (+), SATB2 (-), CDX2 (-), CEA (+), and PSA (-). The pathological diagnosis concluded a malignant tumor in the right occipital lobe, consistent with metastatic SMARCA4-deficient carcinoma, likely originating from SMARCA4-DNSCLC based on morphology and immunohistochemistry. Genetic testing revealed Class II variants with potential clinical significance: ATR: c.6078 + 1G>T (intron 35, VAF: 18.80%); BARD1: p.M420Ifs8 (exon 4, VAF: 16.96%); SMARCA4: p.G256* (exon 5, VAF: 51.82%); and TP53: p.M66Gfs75 (exon 14, VAF: 24.33%); and Level III variants with potential biological significance: NSD1 p.R1700* (exon 14, VAF: 24.33%); microsatellite instability (MSI) was not detected as MSI-H; and tumor mutational burden (TMB) was 27.8 mutations/Mb, indicating high TMB (**Table 1**). Chest contrast-enhanced CT showed scattered solid and ground-glass nodules in both lungs, with the largest in the left lower lobe anterior basal segment (thin-slice Image 205, approximately 23.7 × 14.3 mm), showing mild delayed enhancement on contrast scan, with spiculated and lobulated margins, locally involving the left upper lobe across the interlobar fissure (**Figure 1C**). CT-guided needle biopsy immunohistochemistry results were: CD56 (-), CgA (-), CK (Pan) (+), CK5/6 (-), CK7 (+), CT (-), INSM1 (-), Ki67 (+, 20%), BRG-1 (-) (**Figure 2D**), NapsinA (+) (**Figure 2F**), P40 (-), Syn (-), TTF-1 (+), Vimentin (-), and INI1 (+). The pathological diagnosis confirmed non-small cell carcinoma in the left lower lobe basal segment, consistent with SMARCA4-DNSCLC based on morphology and immunohistochemistry. Combined with pathological and immune marker results, the diagnosis supported SMARCA4-DNSCLC at stage T1cNxM1c IVB. Genetic testing showed identical Class II variants: ATR: c.6078 + 1G>T (intron 35, VAF: 13.35%); BARD1: p.M420Ifs8 (exon 4, VAF: 10.33%); SMARCA4: p.G256* (exon 5, VAF: 27.44%); and TP53: p.M66Gfs75 (exon 4, VAF: 30.23%); no MSI-H detected; and TMB was 34 mutations/Mb, indicating high TMB (**Table 2**). Additionally, truncating mutations in chromatin remodeling genes NSD1: p.R1700* (exon 14, VAF: 23.33%) and CHD8: p.E928* (exon 14, VAF: 6.73%) were identified in the lung sample, categorized as Level III variants with potential biological significance. PD-L1 testing showed TPS <1%, with negative expression in both brain and lung specimens (TPS <1%).

**Table 1:** Summary of pan-solid tumor 437 gene testing for brain samples.

Content of the test	Test results	
Somatic cell variation	A total of 25 somatic cell variants were detected, of which 4 were of definite or potential clinical significance	
Category I: variants with clear clinical significance	not detected	
Class II: Variants of potential clinical significance	ATR:c.6078 + 1G>T	
	BARD1:p.M420Ifs*8	
	SMARCA4:P.G256*	
	TP53:p.M66Gfs*75	
Genetic risk-related variants	not detected	
The NCCN guidelines for this cancer suggest testing for genetic results suggestive of	ALK not detected	BRAF not detected
	EGFR not detected	ERBB2 not detected
	KRAS not detected	MET not detected
	NTRK1 not detected	NTRK2 not detected
	NTRK3 p.L53I	RET p.P799S
	ROS1 not detected	
MINERVA score	inapplicable	
Microsatellite instability (MS)	MSI-H not detected	
Tumor Mutational Burden (TMB)	27.8 mutations/Mb (high TMB)	
Sample quality assessment	eligible	

**Table 2:** Summary of 437 gene testing for pan-solid tumors in lung samples.

Content of the test	Test results	
Somatic cell variation	A total of 30 somatic cell variants were detected, of which 4 were of definite or potential clinical significance	
Category I: variants with clear clinical significance	not detected	
Class II: Variants of potential clinical significance	ATR:c.6078 + 1G>T	
	BARD1:p.M420Ifs*8	
	SMARCA4:P.G256*	
	TP53:p.M66Gfs*75	
Genetic risk-related variants	not detected	
The NCCN guidelines for this cancer suggest testing for genetic results suggestive of	ALK not detected	BRAF not detected
	EGFR not detected	ERBB2 not detected
	KRAS not detected	MET not detected
	NTRK1 not detected	NTRK2 not detected
	NTRK3 not detected	RET not detected
	ROS1 not detected	
MINERVA score	inapplicable	
Microsatellite instability (MS)	MSI-H not detected	
Tumor Mutational Burden (TMB)	34 mutations/Mb (high TMB)	
Sample quality assessment	eligible	

Same methods as in **Table 1**

On September 9, 2024, the patient received postoperative localized intensity-modulated radiotherapy for the brain at doses of GTVp: 51 Gy in 17 fractions of 3 Gy each, and PTV: 51 Gy in 17 fractions of 3 Gy each (**Figure 1D**). During the radiotherapy course, the patient developed Grade 2 radiation-induced oral mucositis, which improved after standardized symptomatic and supportive care. During radiotherapy, concurrent chemotherapy with pemetrexed plus carboplatin was administered, along with bevacizumab to reduce cerebral edema; after radiotherapy, toripalimab infusion was initiated. A review chest CT scan in October 2024 showed slight shrinkage of the left lower lobe anterior basal segment lesion to 19.1 × 11.1 mm (**Figure 1E**), with brain MRI indicating no recurrence, and overall assessment of Stable disease (SD). Subsequently, regular treatment with pemetrexed plus carboplatin, bevacizumab, and toripalimab was given for four cycles. By February 2025, a repeat chest CT showed further slight shrinkage of the left lung lesion to approximately 18.6 × 5.1 mm (**Figure 1F**), with enhanced brain MRI revealing no recurrence; overall, the patient achieved SD. In February 2025, the patient continued with maintenance therapy using bevacizumab plus pemetrexed. In March 2025, radiotherapy for the lung lesion was performed at doses of GTVp: 50 Gy in 20 fractions of 2.5 Gy each, and PTV: 50 Gy in 20 fractions of 2.5 Gy each (**Figure 1G**), followed by maintenance therapy with bevacizumab plus pemetrexed for two cycles. A review chest CT in April 2025 indicated additional slight shrinkage of the left lung lesion to approximately 16 × 7 mm (**Figure 1H**). The patient continued with bevacizumab and pemetrexed maintenance therapy. A follow-up chest CT scan in July 2025 revealed that the mass in the anteromedial basal segment of the left lower lobe had remained largely unchanged (17.8 × 7.9 mm) compared to previous imaging (**Figure 1I**). As of October 2025, the most recent chest CT review in October 2025 showed no significant change in the lesion size at approximately 18.4 × 7.3 mm (**Figure 1J**), with overall assessment of SD.

The treatment strategy for this aggressive, treatment-naïve SMARCA4-DNSCLC case was established by multidisciplinary consensus. The TMB-H status guided the choice of toripalimab plus pemetrexed and carboplatin, notwithstanding PD-L1 negativity, while bevacizumab was used off-label for cerebral edema. This combination proved manageable: only Grade 1 chemotherapy-related toxicities (e.g., fatigue, nausea) occurred, with no Grade 2+ immune-related adverse events, and all were controlled with standard support.

### 3 Methods

Genomic profiling was performed using a commercially available 437-gene next-generation sequencing panel. The assay incorporated rigorous quality controls, including positive controls (cell line and plasmid DNA mixture) and negative controls (healthy human cell line DNA) to monitor reagent and instrument performance throughout the sequencing process. Bioinformatic analysis utilized a proprietary somatic mutation prediction model, developed based on database features and fundamental mutation characteristics.

#### 3.1 Somatic variant calling

Somatic variants were identified by aligning high-quality sequencing reads to the reference genome (hg19) using BWA. Following local realignment and base quality recalibration with GATK, SNVs and indels were called using VarScan2. Detected variants were stringently filtered based on the following criteria: minimum read depth of 20, minimum of 5 variant-supporting reads, strand bias ≤ 10%, exclusion of variants with >1% frequency in the 1000 Genomes or ExAC databases, and removal of recurrent sequencing artifacts using an internal error list. Germline mutations were filtered out by comparison with matched peripheral blood controls.

#### 3.2 TMB calculation

TMB was calculated by summing all somatic base substitutions and insertions/deletions within the coding region of the targeted panel, including synonymous mutations. Known driver mutations (e.g., in “EGFR” and “ERBB2”) were excluded from the count. A minimum variant allele frequency threshold of 2% was applied for mutation calling. The final TMB value was normalized to the size of the panel’s coding region (0.971 Mb) and reported as mutations per megabase (mut/Mb).

#### 3.3 MSI assessment

MSI status was determined by next-generation sequencing of 52 microsatellite loci. When these loci passed quality control, a sample was defined as MSI-H if more than 40% of the loci exhibited instability.

## 4 Discussion

### 4.1 The cooperative chromatin remodeling factor

SMARCA4 (a core subunit of the SWI/SNF complex) influences transcriptional activation and repression by regulating chromatin accessibility. Its inactivating mutations can lead to decreased genomic stability and abnormal activation of oncogenic signaling pathways (e.g., MYC, WNT). In this case, the truncating mutation of SMARCA4 may cause loss-of-function in the SWI/SNF complex, disrupting transcriptional regulation of genes involved in critical cellular processes such as cell cycle control and differentiation. This promotes tumorigenesis and development, manifesting as highly aggressive and metastatic characteristics with elevated tumor mutational burden, consistent with the aggressive phenotypes reported in SMARCA4-deficient tumors (e.g., non-small cell lung cancer, ovarian cancer). CHD family genes and their neighboring genes are primarily involved in DNA repair, cell cycle regulation, and cellular organelle organization. These genes show significant correlations with immune cell infiltration levels, and their mutations may contribute to lung cancer pathogenesis by affecting chromatin structure and gene expression regulation (8). NSD1, a member of the nuclear receptor-binding SET domain (NSD) methyltransferase family, regulates chromatin integrity and gene expression through histone methylation modifications. Its mutations may lead to abnormal histone modifications, subsequently affecting gene expression and chromatin states, potentially contributing to lung cancer development (9, 10). Activation of endogenous retroviral element (ERV) expression that triggers immune evasion (11).

### 4.2 Regarding disrupted DNA damage response pathways

The ATR gene encodes a serine/threonine protein kinase critical for DNA damage repair and cell cycle regulation. BARD1 (BRCA1-associated RING domain protein 1) on chromosome 2q34-q35 interacts with BRCA1 to participate in DNA damage repair and tumor suppression. The tumor suppressor p53 regulates cell cycle control, DNA repair, and apoptosis, with crucial implications for lung cancer progression and prognosis (12). Truncating ATR splice mutations may eliminate its role in stabilizing replication forks, exacerbating replication stress. TP53 deletion allows cells to bypass G1/S checkpoint control, accelerating clonal evolution (12). While literature reports increased PARP inhibitor sensitivity in such patients (ORR 42%) (12), this case did not attempt PARPi therapy. Notably, ATR/BARD1 co-mutations may induce PARPi resistance, requiring combination with ATR inhibitors to overcome (13).

The compelling evidence for a DNA repair-deficient state in this tumor stems from the convergence of systems biology, mutational patterns, and functional genomics. Our protein-protein interaction network analysis established a mechanistic basis for this deficiency, demonstrating that the somatically mutated genes ATR, BARD1, and TP53 reside at central hubs of a network significantly enriched for “Homologous Recombination” and “Fanconi Anemia” pathways, indicating a systemic compromise of the DNA repair machinery. The functional consequence of this compromised network is quantitatively reflected in the high tumor mutational burden observed in both tumor samples. Furthermore, mutational signature analysis, while not revealing the canonical HRD signature SBS3, uncovered a dominant SBS4 signature consistent with tobacco exposure and a substantial contribution from SBS24. This unique profile suggests the activity of a non-canonical mutational process, aligning with the complex molecular context of co-mutations in chromatin remodeling and DDR genes. Together, this multi-faceted genomic evidence robustly supports the existence of a DNA repair-deficient phenotype, solidifying the rationale for future consideration of DNA damage response-targeted therapies.

Key biological effects include: Replication stress tolerance through impaired CHK1 phosphorylation (CHK1 being an ATR-regulated essential kinase), leading to accumulated DNA damage and subsequent tumor cell apoptosis (13); Homologous recombination deficiency caused by BARD1 mutations disrupting BRCA1 complex function and H2A-K15ub modification dynamics (14). Genomic instability from TP53 deletion-mediated acceleration of clonal evolution (15). Clinically, this manifests as PARP inhibitor sensitivity, though ATR/BARD1 co-mutations may necessitate combined ATR inhibitor therapy to overcome potential resistance (13).

### 4.3 Synergistic carcinogenic mechanisms between chromatin remodeling factors and DDR gene mutations

Chromatin remodeling factors (SMARCA4, CHD8, NSD1) play central roles in epigenetic regulation. Notably, DDR gene mutations exhibit potential synergistic effects with chromatin remodeling abnormalities. In this case, the co-occurring mutations in TP53 and BARD1 may lead to dual suppression of the homologous recombination repair (HRR) pathway, synergizing with SMARCA4/CHD8 mutations to exacerbate genomic instability and ultimately drive the TMB-H phenotype. This mechanism finds a parallel in the work of Xue Jing’s team, who demonstrated in pancreatic cancer that epigenetic dysregulation (e.g., SETD2 deficiency) can interact with metabolic reprogramming and potentially impair DDR-related pathways, thereby accelerating tumor progression (16).

### 4.4 Clinical significance of high TMB and immunotherapy potential

The high TMB observed in this case, which we posit stems from the confluence of DDR and chromatin remodeling deficiencies, holds significant clinical implications. Studies indicate that higher TMB correlates with enhanced T-cell recognition, and improved clinical outcomes with ICIs (17). In this case, disease stabilization following toripalimab combination therapy supports the utility of TMB as a biomarker for PD-L1-negative patients, highlighting its critical role in immunotherapy screening (18–20). The elevated TMB in this case may be attributed to: (1) impaired DDR pathway efficiency, (2) transcription-coupled repair (TCR) dysfunction caused by chromatin remodeling abnormalities, and (3) TP53 mutation-associated genomic instability. However, the unique coexistence of TP53 mutation and NSD1 aberration warrants attention. While TP53 mutations may enhance ICI responsiveness via PD-L1 upregulation or immunosuppressive microenvironment induction (21, 22), NSD1 loss could attenuate antitumor immunity by suppressing interferon signaling and modulating the immune microenvironment, potentially promoting immune evasion (9, 10).

### 4.5 Pathological characteristics of the case

Consistent with the aforementioned molecular background, the tumor in this case demonstrated characteristic SMARCA4 loss or diffuse significant attenuation. Epithelial markers (CK(Pan), CK7) exhibited diffuse strong positivity. Lung adenocarcinoma marker TTF-1 was negative in 80%-90% of cases, with weak to moderate expression observed in a minority of cases (23–25). While approximately 80–90% of SMARCA4-deficient undifferentiated tumors are TTF-1 negative, a subset of cases (approximately 10–20%) can exhibit TTF-1 positivity, as demonstrated in the present case. This alerts clinicians and pathologists that when encountering TTF-1 positive tumors, particularly those with atypical morphology, SMARCA4-deficient tumors must be included in the differential diagnosis, and SMARCA4/BRG-1 immunohistochemical staining should be performed to avoid misdiagnosis. Combined with the morphological features of the brain and pulmonary lesions and immunohistochemical results, the findings align with the phenotype of SMARCA4-deficient undifferentiated adenocarcinoma, characterized by heterogeneous expression of TTF-1/NapsinA. Key markers included BRG-1 (-) (indicating SMARCA4 protein loss) and retained INI1 (+) (excluding INI1-deficient tumors).

Notably, immunohistochemical differences were observed between the primary lung lesion and the brain metastasis: The primary lesion retained the differentiation marker NapsinA (+), while the brain metastasis showed NapsinA (-), suggesting dedifferentiation and loss of acinar features in the metastatic site. The Ki67 index increased from 20% in the primary lesion to 30% in the brain metastasis, indicating enhanced proliferative activity and aggressiveness of the metastatic clone. However, BRG-1 (-) was maintained in the metastasis, consistent with stable retention of driver genetic alterations, as supported by molecular testing.

### 4.6 Clarification on SMARCA4 zygosity and functional loss

The genomic profile of this case provides nuanced insight into the mechanism of SMARCA4 deficiency. The identified SMARCA4 p.G256* mutation was present at a variant allele frequency (VAF) of 51.82% in the primary lung lesion and 27.44% in the brain metastasis, a finding consistent with a heterozygous mutation rather than a homozygous deletion. This underscores that the term “SMARCA4-deficient” in a diagnostic context refers primarily to the functional loss of the protein, as definitively demonstrated by the negative BRG1 immunohistochemical stain. The complete absence of BRG1 protein, despite the presence of only one detected truncating mutation, strongly implies the existence of a ‘second hit’ that inactivated the second allele. This second event could be a copy-neutral loss of heterozygosity, a deep intronic splice-site mutation, or an epigenetic alteration not detectable by our targeted DNA sequencing panel. Thus, the phenotypic diagnosis of SMARCA4 deficiency is firmly established by IHC, while the genetic data reveal a complex, likely biallelic, inactivation mechanism.

### 4.7 Genetic mutations, treatment strategy, and outcomes

This case involved a malignant tumor harboring composite mutations in chromatin remodeling factors (SMARCA4, CHD8, NSD1) and DNA damage repair (DDR) genes (ATR, BARD1, TP53), accompanied by high tumor mutational burden (TMB-H). The pathogenic heterozygous SMARCA4 p.G256* truncating mutation, associated with loss of SMARCA4 protein function, is the central genetic alteration underlying the development of this SMARCA4-deficient tumor.

Following surgical resection of the brain lesion (indicated by initial symptoms and imaging findings) and adjuvant intensity-modulated radiotherapy, no recurrence or metastasis was detected in the brain by May 2025. Both brain and lung lesions showed PD-L1 negativity (TPS <1%). Given the lack of targeted therapies for SMARCA4-deficient tumors, which are typically chemotherapy-resistant but immunotherapy-sensitive (median PFS: 7.5 vs. 3.5 months, P<0.001) (26), and considering the potential benefit of high TMB for immunotherapy (27), the patient received toripalimab combined with chemotherapy (aligned with clinical guidelines). Anti-angiogenic agents were incorporated to improve blood-brain barrier permeability and manage cerebral edema (28). After five treatment cycles, the disease remained stable, with a progression-free survival (PFS) of 15 months by October 2025, exceeding the median PFS reported in literature, suggesting a unique therapeutic benefit mechanism in this case. This positive therapeutic response is likely closely related to the patient’s distinctive tumor molecular profile and the comprehensive treatment strategy integrating local and systemic therapies.

The remarkable clinical benefit observed in this case, evidenced by a PFS of 15 months, is likely intricately linked to its distinctive molecular landscape. Firstly, the high tumor mutational burden (TMB-H, 34 mut/Mb) stands out as the most prominent predictive biomarker. This TMB-H status meets the FDA-approved criteria for pembrolizumab use in TMB-H solid tumors and strongly suggests enhanced tumor immunogenicity, which likely served as the primary driver enabling the immune checkpoint inhibitor to exert sustained effects and achieve long-term disease stability (29). Secondly, the deleterious mutations identified in DDR pathway genes (such as ATR and BARD1) provide a plausible biological explanation for the observed TMB-H phenotype (30). The functional loss of these genes leads to increased genomic instability and mutation accumulation, thereby indirectly shaping a tumor microenvironment more favorable for immunotherapy.

The molecular findings in this case carry significant implications for future treatment strategies. Although SMARCA4 deficiency itself currently lacks direct targeted therapies, the accompanying molecular signature of ‘TMB-H + BARD1 inactivation mutation’ provides a clear direction for subsequent therapeutic exploration: upon disease progression, PARP inhibitor therapy could be reasonably considered based on the homologous recombination deficiency (HRD) status suggested by the BARD1 mutation. Concurrently, the high TMB strongly supports the continued rationale for employing immunotherapy strategies. Therefore, comprehensive molecular profiling in such patients is crucial for identifying these potential therapeutic opportunities.

## 5 Clinical implications

This case provides critical clinical insights that can inform current practice. Diagnostically, it underscores the necessity of performing comprehensive DDR gene testing alongside SMARCA4 status assessment, given the high frequency of therapeutically relevant co-mutations. Therapeutically, our experience supports a paradigm shift in treating such complex molecular profiles. It demonstrates that PD-L1-negative, TMB-H tumors with co-mutations in chromatin remodeling and DDR pathways can achieve significant clinical benefit from first-line “immune-chemotherapy” combinations, challenging the conventional, PD-L1-centric immunotherapy selection paradigm. For patients with brain metastases, this case reinforces the importance of integrating anti-angiogenic agents and radiotherapy into the treatment strategy. Furthermore, it highlights the need for caution regarding PARP inhibitor monotherapy in the context of co-existing ATR mutations, suggesting potential intrinsic resistance, and posits that EZH2 inhibitors represent a rational therapeutic avenue for future exploration in SWI/SNF-deficient contexts.

## 6 Future perspectives

Building on the implications of this case, several key directions for future research emerge. First, there is a pressing need to prospectively validate the efficacy of rational combination therapies in larger, molecularly defined cohorts. Clinical trials investigating combinations such as immune checkpoint inhibitors with PARP inhibitors (potentially augmented by ATR inhibition) or with EZH2 inhibitors are strongly warranted. Second, the development of advanced model systems—such as patient-derived organoids or genetically engineered models harboring these specific co-mutations—is crucial to functionally validate the proposed synergistic mechanisms and to serve as preclinical platforms for drug testing. Finally, future efforts should focus on moving beyond single-gene biomarkers to develop integrated diagnostic algorithms. These algorithms would incorporate complex co-mutation patterns, mutational signatures, and pathway-level alterations to better stratify patients for mechanism-driven, multi-targeted therapeutic strategies, ultimately advancing precision immunotherapy for rare NSCLC subtypes.

## 7 Limitations

This study has several limitations that should be considered when interpreting the findings. First, as a single-case report, the insights generated are descriptive and hypothesis-generating; the favorable treatment response observed may not be generalizable to all SMARCA4-DNSCLC and could be influenced by the unique biology of this tumor or the multi-modal therapy itself. Second, the genomic analysis was confined to a targeted ~400-gene panel. While this approach allowed for focused and deep sequencing, it inherently limits the detection of variants in genes outside the panel’s scope, such as those involved in non-canonical cancer pathways or deep intronic regions. Third, while our IHC results conclusively demonstrate the loss of SMARCA4 protein function, the precise genetic mechanism leading to the inactivation of the second allele remains undetermined. Moreover, the absence of matched normal tissue sequencing (e.g., peripheral blood) represents a limitation. While we applied stringent bioinformatic filters to distinguish somatic variants, the lack of a matched normal control precludes the definitive exclusion of rare germline polymorphisms or low-level clonal hematopoiesis of potential clinical significance. The heterozygous VAF of the SMARCA4 truncating mutation suggests a two-hit mechanism, but our targeted sequencing panel was unable to identify the nature of the second hit, which could include structural variants or epigenetic silencing.

Although the SMARCA4 p.G256* mutation was associated with a high VAF consistent with loss-of-function, the absence of comprehensive copy number analysis means we cannot definitively confirm biallelic inactivation. The proposed synergistic mechanisms between chromatin remodeling and DNA damage repair deficiencies, though biologically plausible, remain speculative without functional validation in models. Finally, the concurrent application of multiple treatment modalities (surgery, radiotherapy, chemo-immunotherapy, bevacizumab) makes it impossible to isolate the individual contribution of immunotherapy, which was selected based on the TMB-H status, to the clinical outcome. However, for patients with extensively metastatic stage IV NSCLC, the treatment principle should be comprehensive, requiring the integration and synergy of local and systemic therapies to achieve optimal clinical outcomes, improve quality of life, and prolong survival.

## Data Availability

The original contributions presented in the study are included in the article/supplementary material. Further inquiries can be directed to the corresponding author/s.
